# Comparison between single-segment Isobar EVO dynamic stabilization and Isobar TTL dynamic stabilization in the treatment of lumbar degenerative diseases: a single center retrospective study over 4 years

**DOI:** 10.1186/s12891-022-05913-6

**Published:** 2022-11-19

**Authors:** Jianbin Guan, Tao Liu, Ningning Feng, Guozheng Jiang, Wenhao Li, Kaitan Yang, He Zhao, Yongdong Yang, Xing Yu

**Affiliations:** Dongzhimen Hospital Beijing University of Chinese Medicine, Beijing, China

**Keywords:** Dynamic stabilization, Non-fusion, Lumbar degenerative diseases, Adjacent segment, Degeneration, Isobar

## Abstract

**Objective:**

Posterior instrumented fusion is the most widely accepted surgical treatment for spinal stenosis and disc herniation. However, fusion can affect daily activities due to lumbar stiffness. In recent years, dynamic stabilization has been introduced to overcome the drawbacks of fusion, however, it is inconclusive whether dynamic stabilization requires the maintenance of a level of activity that is closer to the physiological state of activity for better clinical efficacy. The purpose of this study was to compare the effectiveness of dynamic stabilization with different levels of activity (Isobar EVO and TTL) in the treatment of spinal stenosis and disc herniation.

**Methods:**

This study retrospectively reviewed 80 consecutive patients with lumbar degenerative diseases who were undergoing surgical treatment between March 2014 and July 2018. 41 patients (EVO group) and 39 patients (TTL group) underwent fenestrated decompression with Isobar EVO stabilization and Isobar TTL stabilization, respectively. Clinical outcomes, radiographic data, and postoperative complications were compared between the two groups.

**Results:**

At an average follow-up of 52.23 ± 2.97 months, there were no significant differences in the oswestry disability index (ODI) (*P* > 0.05). The visual analog scale for back pain (VAS_back_) and visual analog scale for the leg pain (VAS_leg_) of the EVO group were lower compared with the TTL group (*P* < 0.05). The range of motion (ROM) of operated segments were significantly higher in the EVO group as compared to the TTL group (*P* < 0.05). The intervertebral space height (ISH) of upper adjacent segments were significantly higher in the EVO group as compared to the TTL group (*P* < 0.05). The overall complications were less in the EVO group, but the difference was not statistically significant (*P* > 0.05).

**Conclusion:**

Both Isobar EVO dynamic stabilization and TTL dynamic stabilization can improve clinical outcomes of patients with spinal stenosis and disc herniation. Isobar EVO has advantages over Isobar TTL in terms of improving low back and leg pain, maintaining mobility of the operated segment, and preventing further degeneration of the upper adjacent segment.

## Introduction

Lumbar fusion has been the gold standard for spine stabilization in degenerative, traumatic, pathologic, and deformity conditions, relieving symptoms by decompressing the nerve root of the responsible segment and reconstructing the stability and sequence of the operating segment through fixation and fusion, which can achieve an immediate satisfactory effect. However, fusion alters the normal biomechanical environment of the lumbar spine functional unit, resulting in loss of motion of the surgical segment and accelerated degeneration of the adjacent segment [1, 2]. Dynamic stabilization theoretically eliminates or reduces these risks. Dynamic stabilization procedures include lumbar disk replacement, nucleus replacement, use of interspinous spacers, and pedicle screw-based posterior dynamic stabilization (PDS) [1, 3]. Because motion preservation has been used successfully elsewhere in the body, the potential for motion preservation in the lumbar spine is being debated. Rather than the rigid rods used in standard instrumented fusion, pedicle screw-based dynamic stabilization PDS employs motion-preserving constructs that interconnect the pedicle screw fixation. The Isobar dynamic stabilization system, a popular posterior transpedicular dynamic internal fixation non-fusion stabilization system, provides spinal stability while allowing the surgical segment to move freely. Since its inception, the Isobar dynamic stabilization system has gone through five generations, including Isolock (1993), Isobar TTL (1998), Aladyn (2002), Isobar Duo (2008), and Isobar EVO (2010), and has evolved into a mature internal fixation device. Based on Wolff’s law for fusion surgery, the original design concept of Isobar stabilization system is to promote interbody fusion [4, 5]. And some clinicians have also successfully applied the Isobar dynamic stabilization system to non-fusion technology [6–8]. However, how much range of motion should be retained in dynamic stabilization, how to retain it, and which exercise method is more conducive to maintaining the physiological environment of the lumbar spine and slowing the degeneration of the adjacent vertebrae have always been problems that researchers from all over the world have been working to solve. Given these concerns, we conducted research using the Isobar system. Because TTL and EVO’s activity and volume profiles differ only slightly, it is better suited for investigating the connection between dynamic stability and activity retention. Furthermore, the current literature on the Isobar system is either short-term or only TTL system follow-up, and there is insufficient evidence-based medical evidence on the efficacy of the EVO system and its comparison with the TTL system [9–11].

This study compared preoperative and postoperative imaging as well as clinical indicators of different degrees of activity Isobar dynamic internal fixation system combined with decompression in patients with lumbar degenerative disease to determine whether increasing the dynamic stabilization device’s range of motion moderately would have a more positive impact on patient imaging and clinical outcomes.

## Materials and methods

### Patients

This study retrospectively reviewed 80 consecutive patients with spinal stenosis and lumbar disc herniation who were undergoing surgical treatment with Isobar TTL/EVO between March 2014 and July 2018. Among them, 41 patients (EVO group) had fenestration decompression and Isobar EVO stabilization, and 39 patients (TTL group) underwent fenestration decompression and Isobar TTL stabilization. This study has been approved by the Ethical Committee of the Dongzhimen Hospital affiliated to Beijing University of Chinese Medicine (2022DZMEC-085-04). Formal consent for the inspection of patients’ photos and medical records is not necessary for this kind of study. Additionally, the study was carried out in accordance with the moral guidelines that were established by the Declaration of Helsinki and its following amendments. Inclusion criteria were as follows: (1) combined with degenerative changes such as spinal stenosis and disc herniation (Herniation of the L3/4, L4/5, L5/S1 disc or severe herniation involving more than half of the spinal canal); (2) no improvement after 3-months of conservative treatment; (4) underwent the operation of dynamic stabilization (Isobar TTL or EVO, Scient’x-Alphatec, France); and (5) with complete clinical and imaging data, and with a follow-up of more than 48 months. Exclusion criteria were as follows: (1) with more severe lumbar instability (lumbar spondylolisthesis ≥ II degree), severe scoliosis or rotational deformity and other lumbar spine diseases; (2) a history of lumbar surgery; (3) with lumbar fractures, tumors, infections, ankylosing spondylitis, etc.; (4) with hip disease, cervical spondylotic myelopathy, or other illnesses that impacted the assessment of the therapeutic outcome; (5) severe osteoporosis (T value ≤ − 2.5 with single or multiple fragility fractures or T value ≤ − 3.0).

### Instrument

The Isobar TTL and EVO dynamic fixation devices manufactured by Scient’x-Alphatec in France. The difference between the two is that the EVO system’s flexion and extension activity is increased from 2.25° to 4.5° when compared to the TTL system. The longitudinal displacement was increased from 0.2 mm to 0.8 mm, the dynamic rod curvature was increased from 8° to 12°, and the titanium ring profile was reduced by 25%.

### Surgical procedure

All patients underwent general anesthesia in the prone position. Autologous blood transfusion was used during the operation. In each group, interlaminar fenestration decompression was performed through the posterior median approach at the responsible levels. The sacrospinous muscles on both sides were stripped to expose the surgical segment’s spinous process, lamina, and lateral sides of the facet joints. And we did our best to protect the joint capsule of each surgical segment’s facet joint during this procedure. The C-arm was used to confirm that the surgical segment was correct, and then universal pedicle screws of appropriate length and thickness were sequentially installed. And the screw tip should point as far to the upper end plate of the vertebral body as possible. Remove some spinous processes, laminae, hyperopic osteophytes, and hypertrophic ligamentum flavum from the stenotic segment of the lumbar spine with rongeurs and lamina forceps. During this procedure, we did our best to avoid damage to the inside of the facet joint. Submerged decompression of the lateral recess was used until the compression of the nerve root canal and central spinal canal is completely relieved, and then the prolapsed nucleus pulposus tissue were explored and removed. If the intervertebral disc with inclusive herniation does not compress the nerve root, it should be left alone to avoid disturbing the surgical segment’s intervertebral space. TTL or EVO dynamic rods and locking bolts were implanted according to the fixed signs after sufficient decompression. The incision was closed layer by layer after adequate hemostasis, flushing, and an indwelling epidural drainage tube were performed. Antibiotics were routinely administered 24 hours after surgery to prevent infection, and the drainage tube was removed 24–48 hours later depending on the drainage volume. Wear the brace 3 to 5 days after the operation to help patients get out of bed while gradually retraining lower back muscles. The brace is typically worn for 1 month following surgery. Following the removal of the brace, the patients is given functional exercise recommendations and instructed to exercise their low back on a regular basis.

### Clinical and radiological evaluation

Outpatient re-examinations were performed on all patients. X-ray films of the lumbar spine in the anterolateral, hyperextension and hyperflexion positions, as well as a lumbar spine MR examination, were taken 1 month, 1 year, and every year after the operation.

Clinical outcomes were assessed through the visual analog scale (VAS) for back and leg pain, the oswestry disability index (ODI). Posteroanterior, lateral, and dynamic radiographs with flexion and extension views were obtained preoperatively and at the last follow-up.

**Fig. 1:**
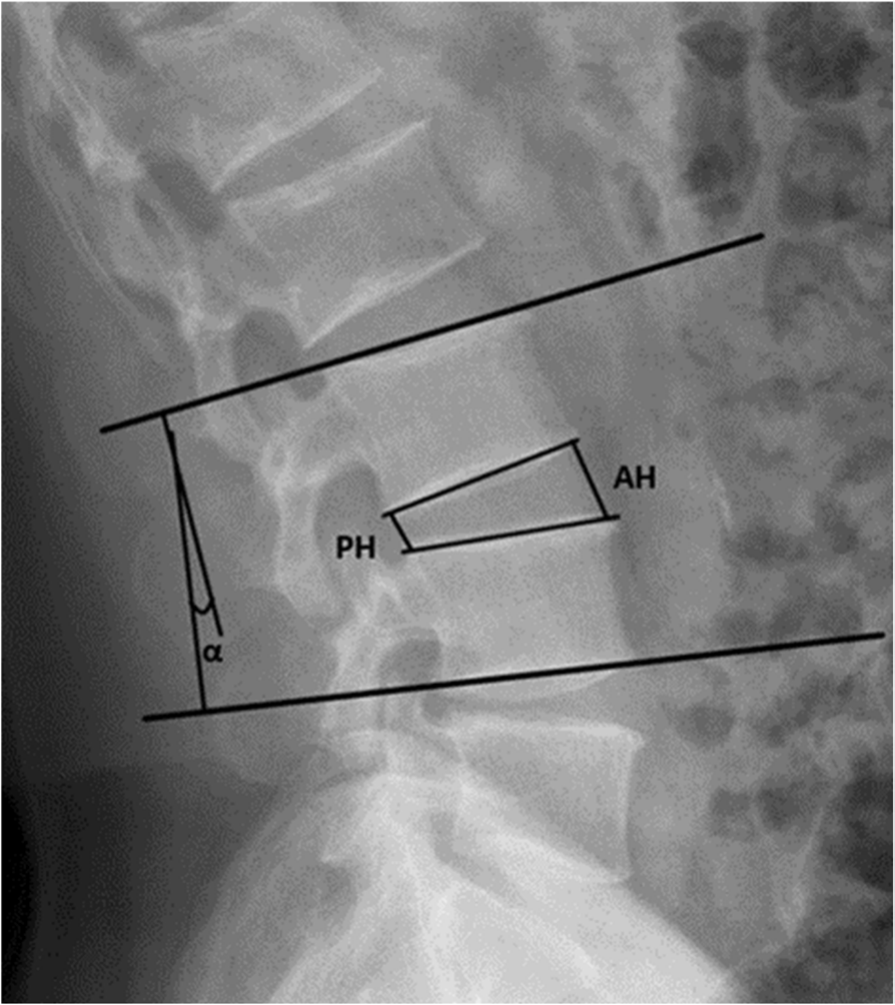
Intervertebral space height (ISH) The height of the intervertebral space is expressed by the mean value of the anterior height (AH) and the posterior height (PH) of the vertebral body on the lateral X-ray of the lumbar spine. Range of motion (ROM) The lumbar vertebral range of motion was indicated by the Cobb angle α of the operative segment and the adjacent segment. The Cobb angle α of the lumbar vertebrae was measured in the lateral, hyperextension, and hyperflexion positions. For the L5-S1 ROM, the upper endplate of the L5 vertebral body and the upper endplate of the sacrum were selected for measurement

Because adjacent segment disease (ASD) frequently occurs in the upper adjacent segment of the surgical segment, the imaging indicators of the upper adjacent segment were only evaluated in this study [12]. A “double halo sign” (radiolucent line around the implant > 2 mm wide) on X-rays was defined as screw loosening [13]. X-ray films of the lumbar spine were taken in the lateral, hyperextension, and hyperflexion positions during the follow-up, and the intervertebral heights of the operative segment and the adjacent segment were measured. The average height of the anterior and posterior borders of the intervertebral space on lumbar lateral X-ray films before and after each follow-up was used to calculate intervertebral height (Fig. 1). The intervertebral height of the adjacent vertebra can further evaluate the degeneration of the intervertebral disc by measuring the intervertebral height of the surgical segment to assess whether there are surgical complications such as endplate collapse, whether the intervertebral foramen has been indirectly decompressed, and whether the relationship between the normal physiological curvature of the lumbar spine and the force line has been restored. Evaluate the Cobb angle of the operative segment and adjacent segments on the lumbar spine in front, lateral, and hyperextension and hyperflexion radiographs to assess lumbar range of motion (Fig. 1). The degeneration of adjacent segments was evaluated using the University of California at Los Angeles (UCLA) system. Preoperative and postoperative sagittal median T2WI were used in the MR examination. The system’s key evaluation indicators were intervertebral space height, osteophyte formation, and endplate sclerosis.

### Statistical analysis

SPSS 19.0 software was used for statistical analysis. Continuous variables are expressed in the form of mean ± standard deviation. Two independent samples t-tests were used to compare differences between groups, and paired samples t-tests were used to compare differences between preoperative and final follow-up. To compare differences between groups, enumeration data and grade data (the UCLA system) were compared using the chi-square test. When *P* < 0.05, the difference was considered to be statistically significant.

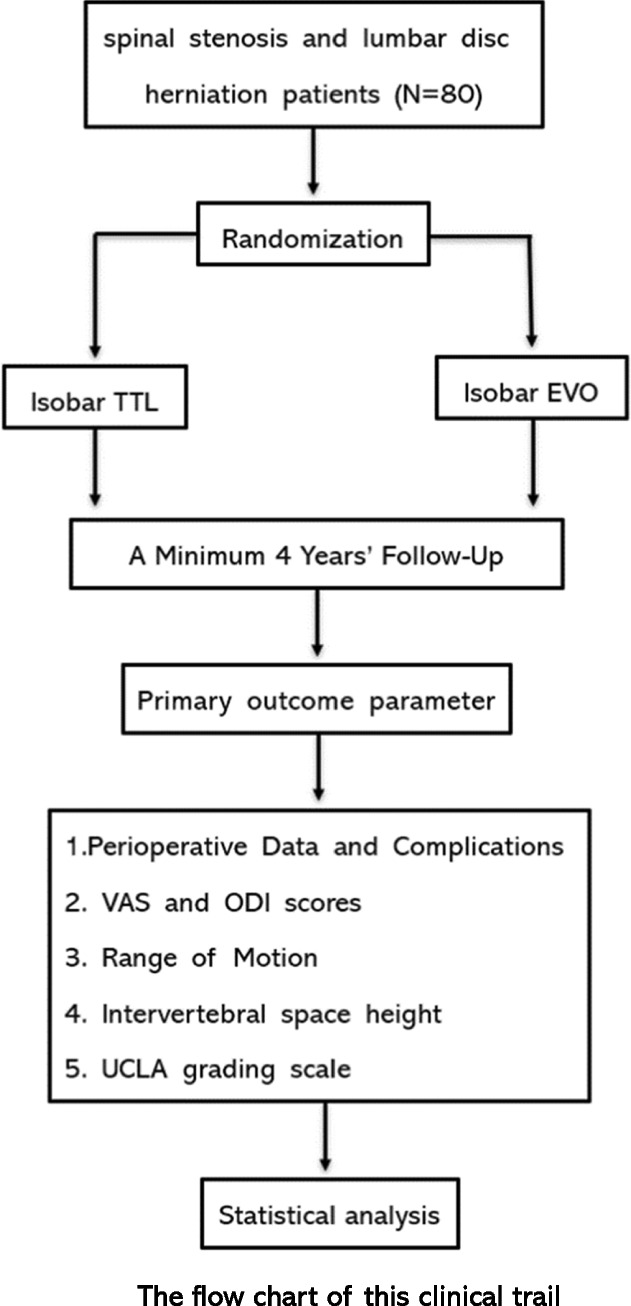



## Results

### Perioperative data and complications

There were no statistically significant differences between the EVO group and the TTL group in age, sex, the distribution of surgical segments, follow-up time and type of disease (*P* > 0.05, Table 1).

**Table 1:** Comparison of demographic and clinical characteristics of 2 groups of patients who underwent TTL or EVO

	TTL (*n* = 39)	EVO (*n* = 41)	*P*
Age	46.03 ± 9.46	46.27 ± 8.72	0.78*
Gender (male/female)	19/20	17/24	0.51†
Follow-up time (months)	52.05 ± 2.79	52.39 ± 3.15	0.67*
Operative segment			0.88†
L3/4	3	4	
L4/5	23	25	
L5/S1	13	12	
Diseases			0.67†
Spinal stenosis	21	24	
Lumbar disc herniation	18	17	
Complication n, (%)	6, (15.4%)	2, (4.9%)	0.117†

Values are presented as mean ± standard deviation

*P* values are based on the Independent two-sample t-test * or chi-square test†

In the EVO group, two patients developed transitory radiating pain after surgery, which was alleviated by medication. The mean follow-up duration was 52.39 ± 3.15 months (range, 48-57 months). There were no cases of screw loosening, incision infection, screw misplacement, screw breakage, or reoperation.

In the TTL group, four patients developed transitory radiating pain after surgery, which was relieved by medication. One patient developed severe low back pain, which was alleviated by nonsteroidal anti-inflammatory drugs (NSAIDs). 53 months following surgery, one patient occurred upper adjacent degeneration disease. Surgery was used to remove the Isobar TTL, and associated surgical procedures were also performed on the upper adjacent vertebra. Recovery from surgery went well. The mean follow-up duration was 52.05 ± 2.79 months (range, 49-56 months). There were also no cases of screw loosening, incision infection, screw misplacement, or screw breakage.

The incidence of complications was lower in the EVO group than in the TTL group (4.9% vs. 15.4%), but the difference was not statistically significant (*P* = 0.117; Table 1).

### Clinical outcomes

The VAS and ODI scores improved significantly at the last follow-up in both the groups (both *P* < 0.05). There was no statistically significant difference in VAS_back_, _leg_ and ODI scores between the two groups preoperative. However, the VAS_back_ and VAS_leg_ scores at the last follow-up, the were lower in the EVO group than that in the TTL group, and the difference was statistically significant (*P* < 0.05; Table 2).

**Table 2:** Clinical outcomes

	TTL (*n* = 39)			EVO (*n* = 41)		
	ODI%	VAS (Back)	VAS (Leg)	ODI%	VAS (Back)	VAS (Leg)
Pre-operation	66.34 ± 9.65	6.18 ± 1.02	6.62 ± 1.66	63.18 ± 7.45	6.39 ± 0.74	6.87 ± 1.12
Final follow-up	11.83 ± 3.71	1.13 ± 0.73†	1.13 ± 0.80†	10.76 ± 3.62†	0.80 ± 0.71*†	0.78 ± 0.65#†
*P*	< 0.01	< 0.01	< 0.01	< 0.01	< 0.01	< 0.01
P*	0.104	0.238	0.861			
*P*#				0.199	0.049	0.041

Data are presented as mean ± standard deviation

ODI, Oswestry disability index; VAS, visual analogue scale

*Significant difference preoperatively follow-up between the TTL and EVO groups using the Independent two-sample t-test, *P* < 0.05

#Significant difference at the final follow-up between the TTL and EVO groups using the Independent two-sample t-test, *P* < 0.05

†Significant difference between pre- and post-operative condition in each group using the paired t-test, *P* < 0.05

### Radiological outcomes

#### Range of motion

**Fig. 2:**
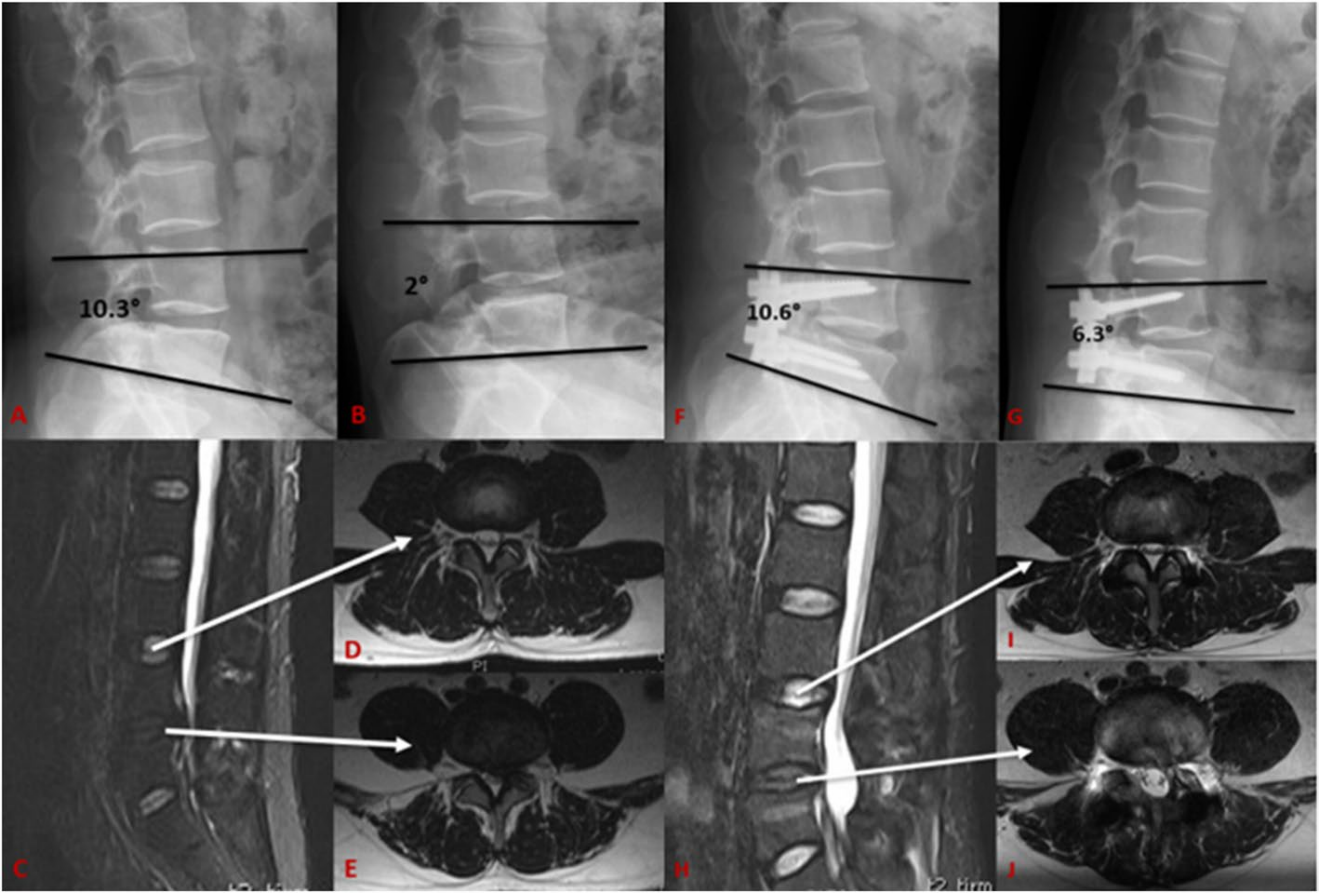
A 55-year-old man had spinal stenosis and disc herniation at L3–4, and the ROM of the surgical segment was 8.3° based on X-ray (**A**-**B**). The MRI of pre-operation showed that the disc signal of L3/4 and L4/5 (**C**-**E**). He underwent fenestration decompression and posterior EVO dynamic fixation at L4–5. The radiographs obtained 53 months after the operation showed the operative segment’s ROM was 4.3°, and the ISH of the L4/L5 remained unchanged from preoperative. The L3/L4 and L4/5 intervertebral disc signal was normal, with no significant change from preoperative (**F**-**J**)

**Fig. 3:**
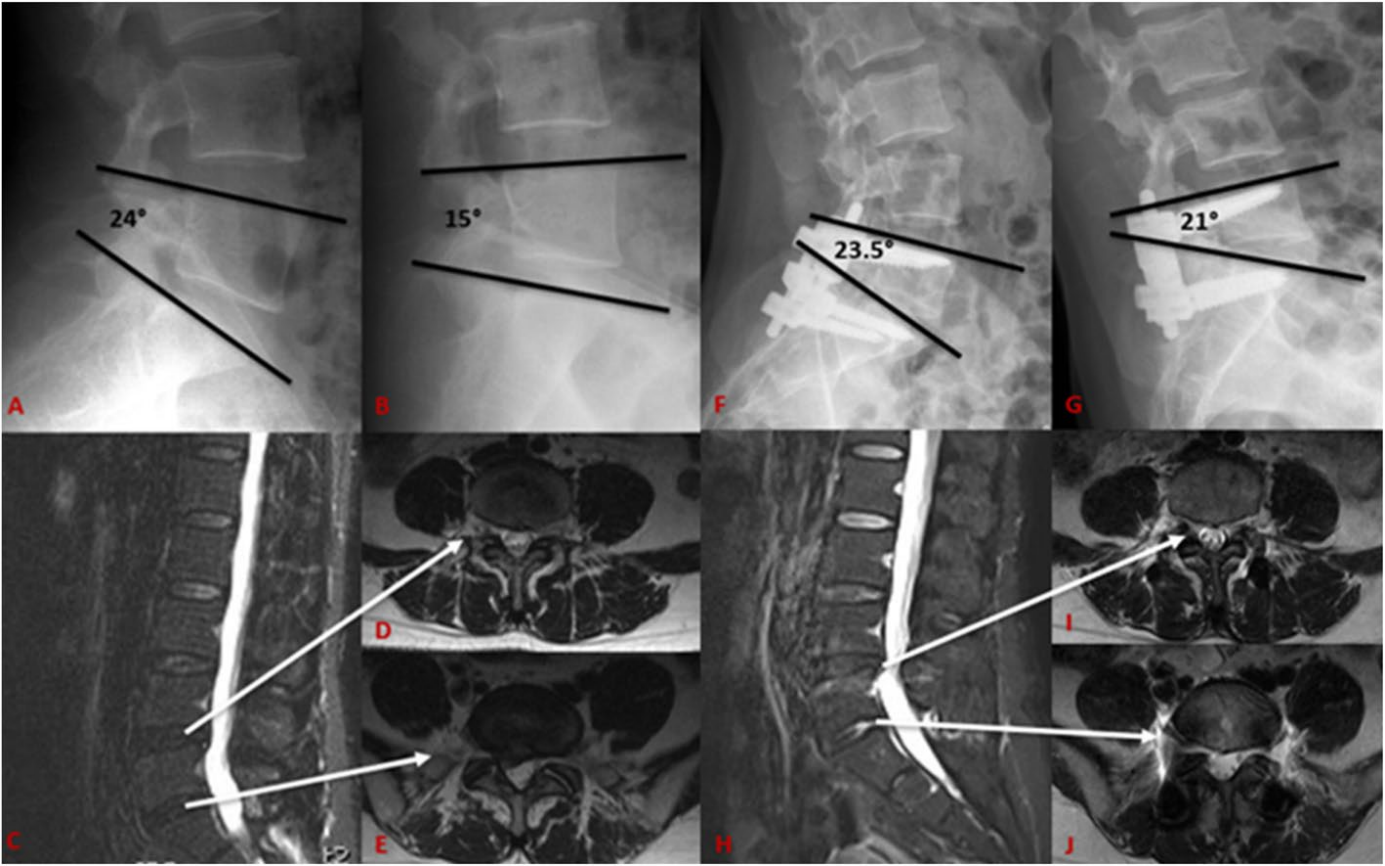
A 48-year-old woman had disc herniation at L5–S1, and the ROM of the surgical segment was 9° based on X-ray (**A**-**B**). The MRI of pre-operation showed that the disc signal of L4/5 and L5/S1 (**C**-**E**). She underwent fenestration decompression and posterior TTL dynamic fixation at L5-S1. The radiographs obtained 49 months after the operation showed the operative segment’s ROM was 2.5°, and the L4/5 ISH was slightly lost compared to preoperative. The L5/S1 ISH did not change significantly compared to preoperative, the L4/L5 disc signal was good, but there was slight bulging, the degree of spinal stenosis did not increase significantly compared to the pre-operation, and the UCLA classification changed from grade I to grade II. And there was no significant difference in the L5/S1 intervertebral disc signal from before surgery (**F**-**J**)

There were no significant differences in the mean ROM values of the operative segment and the upper adjacent segment between the two groups preoperatively. However, the mean ROM values of the operative segment and the upper adjacent segment at the final follow-up were significantly lower than that at pre-operation between the two groups (*P* < 0.05). And the ROM at operative segments of the EVO group were significantly higher than that of the TTL group (4.23° ± 0.42° vs. 2.16° ± 0.56°) at the final follow-up (*P* < 0.05; Table 3). The Typical cases of Isobar EVO and Isobar TTL were showed in Figs. 2 and 3.

**Table 3:** Radiological outcomes

Groups	Pre-operation	Final follow-up	*P*
ROM of operative segment(°)			
TTL	8.33 ± 0.89	2.16 ± 0.56*†	< 0.01
EVO	8.19 ± 0.84	4.23 ± 0.42*†	< 0.01
*P*	0.478	< 0.01	
ROM of upper adjacent segment(°)			
TTL	8.09 ± 0.50	8.14 ± 0.48	0.063
EVO	8.04 ± 0.72	8.10 ± 0.66	0.110
*P*	0.754	0.748	
ISH of operative segment(mm)			
TTL	11.07 ± 1.65	11.47 ± 0.86	0.144
EVO	11.59 ± 0.83	11.51 ± 0.59	0.665
*P*	0.078	0.807	
ISH of upper adjacent segment(mm)			
TTL	11.59 ± 0.67	10.98 ± 0.63*†	< 0.01
EVO	11.19 ± 1.60	11.33 ± 0.67*	0.633
*P*	0.162	0.019	

ROM, range of motion; ISH, intervertebral space height

*Significant difference preoperatively and at the final follow-up between the TTL and EVO groups using the Independent two-sample t-test, *P* < 0.05

†Significant difference between pre- and post-operative condition in each group using the paired t-test, *P* < 0.05

#### Intervertebral space height

Preoperatively and at the final follow-up, there were no significant differences in the mean ISH values of the operative segment and upper adjacent segment between the two groups. However, the ISH values at the upper adjacent segment of the EVO were significantly higher than that of the TTL group (11.33 ± 0.67 mm vs. 10.98 ± 0.63 mm) at the final follow-up (*P* < 0.05; Table 3).

#### UCLA grading scale

According to UCLA systematic evaluation criteria, one and six of the 39 TTL and 41 EVO groups, respectively, had imaging manifestations of adjacent vertebral degeneration, and the difference was statistically significant (*P* = 0.018). In the upper adjacent segment of the TTL group, five cases of UCLA grade 1 changes and one case of UCLA grade 2 changes occurred, with statistically significant differences between preoperative and final follow-up (*P* = 0.049, Fig. 3). There was one case of UCLA grade 1 change in the EVO group’s upper adjacent segment, and the difference between preoperative and final follow-up was not statistically significant (*P* < 0.05, Table 4, Fig. 2).

**Table 4:** UCLA grading scale

	Pre-operation				Final follow-up			
	I	II	III	IV	I	II	III	IV
TTL (*n* = 39)	20	19	0	0	14	19	5	1
EVO (*n* = 41)	23	18	0	0	22	19	0	0
X2	0.186				10.060			
*P*	0.666				0.018			

*P* values are based on chi-square test

## Discussion

### Mechanism of Isobar dynamic stabilization system

The lumbar spine dynamic stabilization internal fixation technique aims to effectively maintain spine stability while preserving a certain physiological mobility of the implanted segment of the spine [14]. Furthermore, the Isobar Dynamic Stabilization System has the advantage of limiting abnormal lumbar motion in the operative segment and reducing excessive mechanical load on the posterior lumbar column structure while maintaining a certain degree of lumbar motion in the operative segment and, to some extent, avoiding abnormal stress patterns in the adjacent segments, thereby effectively reducing the incidence of postoperative degeneration in the adjacent segments. This system’s operative segment load transfer center is close to the anterior mid-spine column, similar to the physiological state, and the system is subjected to less compressive stress than conventional rigid fixation devices while still allowing the surgical segment to be subjected to a certain stress load on the intervertebral disc [15–17].

### Comparison of EVO dynamic fixation and TTL dynamic fixation

Compared to other dynamic fixation systems such as Dynesys and Bioflex, Isobar’s relatively fixed mobility (TTL 2.25°, EVO 4.5°) makes it more suitable for exploring the relationship between mobility and clinical and imaging studies. The clinical comparison between Isobar dynamic fixation nonfusion and lumbar fusion is a valid measure of clinical efficacy of the Isobar dynamic fixation system. We previously compared the clinical and imaging outcomes of TTL dynamic fixation nonfusion and fusion by meta-analysis [18]. According to the results of this meta-analysis, we found that TTL non-fusion can effectively improve the VAS score and ODI index when compared to fusion, and it has significant advantages in terms of reducing operative time and intraoperative bleeding. The TTL system can maintain normal overall lumbar spine mobility and has a lower incidence of ASD, indicating that the Isobar dynamic stabilization system has advantages in early and mid-term functional recovery and postoperative complications after lumbar spine surgery. However, there are no reports on how much mobility Isobar dynamic fixation retains in vivo, whether the stability of dynamic fixation is affected or other complications arise after appropriate increases in mobility and volume reduction with the EVO system, and there is a lack of retrospective experience with studies comparing the difference in clinical outcomes between single-segment EVO and TTL systems [9, 19]. Our data showed the incidence of complications in the EVO group was lower than that in the TTL group (4.9% vs. 15.4%), although the difference was not statistically significant.

This study retrospectively investigated the clinical efficacy of dynamic fixation of Isobar nonfusion with different activity levels in the treatment of single-segment lumbar degenerative disease at mid- and long-term follow-up by ODI and VAS scores. The ODI scores and VAS_back, leg_ scores in both groups improved significantly before surgery and at the last follow-up, and the differences in VAS_back_, _leg_ scores between the two groups at the last follow-up were statistically significant, suggesting that both methods are more effective in the treatment of single-segment degenerative lumbar spine, and the EVO system is more effective than the TTL system in improving low back and leg pain.

The preservation of mobility is the focus of research on dynamic fixation systems, and one of the main objectives of the Isobar Dynamic Stabilization System applied to the treatment of degenerative lumbar spine disease is to reduce compensatory mobility in the adjacent phases in order to minimize the incidence of ASD. According to related finite element studies, Isobar can combat ASD due to its mechanical properties [6, 20, 21]. However, there is still much debate as to whether this procedure can actually reduce the incidence of ASD in clinical practice and the medium- to long-term clinical efficacy of this procedure [19, 22]. In this study, we found that although the TTL system was able to preserve 2.16° ± 0.56° of the surgical segments in vivo, while the EVO system preserved 4.23° ± 0.42°, the difference between the two groups at the last follow-up of the activity of the upper adjacent segment was not statistically significant, which is not consistent with the findings in previous finite element studies [20, 21]. We conclude that one possible explanation for the insignificant increase in mobility in the adjacent segments of early dynamic fixation is that compensatory mobility is compensated by the entire lumbar spine rather than a single adjacent spine, and that differences in mobility of adjacent spines with different mobility Isobar systems may be reflected with increasing years of follow-up.

The degree of UCLA grading scale in the upper adjacent spine was higher in the TTL group than in the EVO group, in which only two cases of recurrent low back pain and imaging of adjacent segment degeneration occurred in the EVO group at more than 4 years of follow-up, whereas five cases of recurrent low back pain and imaging of adjacent segment degeneration occurred in the TTL group, and one case of clinical symptoms of adjacent spondylosis was treated further in the adjacent segment. In terms of the degree of disc degeneration and the need for re-operation, the change in ISH of the adjacent segments in patients implanted with the EVO system was not statistically different from that before surgery (11.33 ± 0.67 mm, *P* = 0.633), and the degree of disc degeneration was relatively low. Therefore, this study suggests that an EVO system with moderately increased mobility may facilitate disc rehydration in fixed segments to reduce the ability of adjacent segments to carry compensatory loads and may be more effective in preventing disc degeneration in adjacent segments to reduce the incidence of ASD.

In this study, there were no broken nails and rods or screw loosening in either group after surgery, indicating that EVO did not cause new complications while reducing the volume and improving the mobility of the device and previous reports for several reasons. Firstly, fenestration decompression causes less damage to the lumbar spine’s stability. Then, titanium rings and connectors can distribute stress on the implants. Finally, patients with severe osteoporosis were excluded from participating in this study.

## Conclusions

This study demonstrates both Isobar EVO dynamic stabilization and TTL dynamic stabilization can improve clinical outcomes of patients with spinal stenosis and disc herniation. However, the EVO is more advantageous in improving low back pain and leg pain, maintaining mobility of the operated segment, and preventing further degeneration of the upper adjacent segment. Therefore, we believe that the EVO system can replace the TTL system for the treatment of lumbar disc herniation and lumbar spinal stenosis. Nonetheless, larger and longer-term studies are needed to establish the long-term safety and efficacy of dynamic stabilization.

## Limitations

Firstly, as a single-center retrospective study, the choice of surgical procedure relies to a certain extent on the operator’s experience in predicting this type of disease, and the team will further improve the surgical indications and implement them strictly. Second, the follow-up time is relatively short, and it is not possible to assess the long-term clinical efficacy after surgery more comprehensively, and it is necessary to increase the follow-up time for further in-depth study. Finally, the number of cases in this study is relatively small, and there is a possibility of selective bias, which may cause errors in the results.
